# Retraction Note: Prosthetic rehabilitation of patients with maxillary oncology defects using zygomatic implants

**DOI:** 10.1186/s40729-026-00704-3

**Published:** 2026-07-13

**Authors:** Davit Mathevosyan, Sergo Hovhannisyan, Karen Mashinyan, Levon Khachatryan, Aram Badalyan, Gagik Hakobyan

**Affiliations:** 1 Department of Oral and Maxillofacial Surgery, Yerevan State Medical University After M. Heratsi, Yerevan, Abovyan, Armenia; 2 Department of Prosthodontics, Yerevan State Medical University After M. Heratsi, Yerevan, Armenia; 3 Department Head and Neck Surgery Modern Implant Medicine, Yerevan, Armenia; 4 Department Head and Neck Surgery, NAIRI Medical Center, Yerevan, Armenia

Retraction to: International Journal of Implant Dentistry (2024) 10:31 10.1186/s40729-024-00545-y

The Editors-in-Chief have retracted this article.

Following the publication of the article, concerns have been raised regarding irregularities in the content of this article, such as the number of patient samples analysed, the total number of zygomatic implants, and data related to the health-related quality of life. An investigation by the Publisher has noted that the peer review was not carried out in line with the Journal’s policies. The authors were contacted for an explanation for raised concerns; however, they have not responded.

The Editors-in-Chief therefore have lost confidence in this study.

None of the authors has responded to correspondence regarding this retraction.

